# Abnormal Gray Matter Structural Covariance Networks in Children With Bilateral Cerebral Palsy

**DOI:** 10.3389/fnhum.2019.00343

**Published:** 2019-10-11

**Authors:** Heng Liu, Haoxiang Jiang, Wenchuan Bi, Bingsheng Huang, Xianjun Li, Miaomiao Wang, Xiaoyu Wang, Huifang Zhao, Yannan Cheng, Xingxing Tao, Congcong Liu, Ting Huang, Chao Jin, Tijiang Zhang, Jian Yang

**Affiliations:** ^1^The Key Laboratory of Biomedical Information Engineering, Ministry of Education, Department of Biomedical Engineering, School of Life Science and Technology, Xi’an Jiaotong University, Xi’an, China; ^2^Department of Diagnostic Radiology, The First Affiliated Hospital of Xi’an Jiaotong University, Xi’an, China; ^3^Medical Imaging Center of Guizhou Province, Department of Radiology, The Affiliated Hospital of Zunyi Medical University, Zunyi, China; ^4^School of Pharmaceutical Sciences, Shenzhen, China; ^5^School of Biomedical Engineering, Health Science Center, Shenzhen University, Shenzhen, China; ^6^Shenzhen University Clinical Research Center for Neurological Diseases, Shenzhen, China

**Keywords:** cerebral palsy, children, magnetic resonance imaging, cortical thickness, structural covariance network

## Abstract

Bilateral cerebral palsy (BCP) is a common movement disorder in children, which often results in lifelong motor disability. One main symptom of BCP is the limitation of hand function in everyday activities. However, the neuroanatomical basis of this prominent hand impairment is yet to discover. Recent advances mainly focus on the lesions of BCP, but the views on the atypical development of cortical parcellations are extremely lacking. Here, in our study, neuroimaging with network analysis was employed to evaluate the changes of structural covariance networks (SCNs) in BCP children. We aimed to elucidate the alteration of SCNs based on cortical thickness (CT), and to reveal the relationship of CT and hand function in the participants with BCP. SCNs were constructed using covariance between regional CT, which was acquired from T1-weighted images of 19 children with BCP and 19 demographically matched healthy controls (HCs). Compared with HCs, BCP children showed increased CT in several regions involving the bilateral areas (lateral occipital, lingual, and fusiform) and right areas (cuneus, pericalcarine, inferior temporal, middle temporal, superior temporal, and insula). Decreased CT was found in the left superior temporal and right superior parietal cortices. Global network analyses revealed significantly decreased normalized clustering and small-worldness in the BCP network. The area under the curve (AUC) of global network measures varied slightly between the BCP and HC networks. The resistance of the both SCNs to the target and random attack showed no significant difference. Also, the BCP foci (right superior temporal and subtemporal cortex) showed a significantly negative correlation between the CT and manual ability. In this work, we identified the CT-based SCNs changes in children with BCP. The abnormal topological organization of SCNs was revealed, indicating abnormal CT, incongruous development of structural wiring, destructive nodal profiles of betweenness, and moved hub distribution in BCP children. This may provide a neuroanatomical hallmark of BCP in the developing brain. Therefore, our results may not only reflect neurodevelopmental aberrations but also compensatory mechanisms.

## Introduction

Cerebral palsy (CP) describes impaired motor and sensory disorders caused by a brain lesion. The wide array of these disorders is due to the heterogeneous nature of the underlying cerebral lesions (Rosenbaum et al., 2007). These lead to a great burden on families and society (Rosenbaum et al., 2007). Bilateral cerebral palsy (BCP) is the most common type, affecting more than 70% of children with CP (Colver et al., 2014). BCP children are hypertonic, with tight and stiff muscles. The affected joints often gradually become rigid and difficult to move, thus BCP children often exhibit upper limb weakness. The prognosis of upper limb function in BCP children remains unclear, especially for hand ability. Therefore, the understanding of the neuroanatomical and connectional infrastructure for upper limb weakness in BCP children might help to generate opportunities for primary prevention or provide the valuable intervention strategies.

BCP involves injury in the brain’s development. Neuroimaging studies have consistently shown abnormalities in more than 80% of children with BCP (Novak et al., 2017). Periventricular white matter injury (PWMI), easily seen on conventional magnetic resonance imaging (MRI), is the leading finding in these affected children. The structural white matter damage could affect distant tracts like the superior cerebellar peduncle, optic nerves and pathways, and long associative fasciculi that could be conducive to the understanding of the clinical impairment pattern in CP. Besides white matter, cerebellum and gray matter volumes were also significantly reduced in CP patients, which suggested neuronal degeneration and damage (Kulak et al., 2016). The diminished connectivity in the motor and sensory pathways could be an indication in the pathophysiological mechanism for motor dysfunction in BCP patients (Papadelis et al., 2014). Significant differences in morphological features of cortical thickness (CT), cortical curvature, and sulcal depth were found to be associated with the function of motor, cognition, vision and communication abilities (Pagnozzi et al., 2016). The involvement of semi-quantitative structural MRI scoring could help to demonstrate the correlation of sensorimotor function and aberrant structural connectivity of hemiplegia (Fiori et al., 2015). In periventricular leukomalacia (PVL) patients, the cortical volume of the pre- and post-central gyri and the paracentral lobule was negatively correlated with motor ability. And the motor cortical connectivity was decreased within the bilateral somatosensory cortex, cingulate motor area, visual cortex and paracentral lobule in the PVL patients (Lee et al., 2011). Various alterations in brain structure could result in multi-systemic neurobiological abnormalities in BCP patients. These abnormalities might affect large-scale brain networks.

Covariance network analysis of cortical morphology provides a non-invasive tool to probe large-scale brain microstructure abnormalities (Lerch et al., 2006; He et al., 2007, 2008; Alexander-Bloch et al., 2013; DuPre and Spreng, 2017; Xu et al., 2017; Jiang et al., 2018). Such researches have promoted the quantification of anatomical links among cortical parcellations based on inter-regional covariation of various morphometric features, such as CT. A vital assumption underlying structural covariance networks (SCNs) is that the morphological characteristics of inter-areal gray matter would covary since they share common development, maturation and disease propagation effects (Raznahan et al., 2011; Alexander-Bloch et al., 2013; DuPre and Spreng, 2017; Liu et al., 2019). It has also been reported that SCNs correspond with anatomical and functional networks constructed through white matter tractography (Hosseini et al., 2016; Bruno et al., 2017).

Although there are a few neuroimaging studies, the etiology of BCP remains elusive. And the SCNs in BCP children remain unexplored. Moreover, given that CP has recently been reported to be reorganized in cortical pathologies (Papadelis et al., 2018), such as in the somatosensory cortex, SCNs analysis based on cortical morphology may provide new insights into the pathophysiology of this disease. Therefore, insight from a connectional perspective, rather than brain localization, would provide additional information to advance the understanding of organizational features in motor tissues or the underlying physiopathology mechanisms in children with BCP. We hypothesized that common and distinct structural connectivity patterns may exist in BCP children.

To test our hypothesis, 19 children with BCP and 19 healthy controls (HCs) were included. The changes in CT and topological abnormalities of SCNs were investigated. Group comparisons were then conducted for SCNs, and the relationship between structural connection and motor-related clinical measurement in BCP was also examined.

## Materials and Methods

### Participants

The present study included 19 children with BCP (12 males, seven females). The participants met the criteria: (a) diagnosis of BCP with a history of hypoxic injuries associated with labor and delivery; and (b) description of PWMI on a MR imaging report. In addition, each child was classified according to the Gross Motor Function Classification System (GMFCS; Palisano et al., 2008). Moreover, the Manual Ability Classification System (MACS) was applied to classify how BCP children use their hands when handling objects in daily life (Eliasson et al., 2006, 2017).

For the HC group, another 19 children (13 males, six females) with no abnormal history on MR imaging, no neurologic or psychiatric illness, no learning disabilities, and no visual or hearing loss were included.

Demographic details and clinical assessment of the subjects are shown in Table 1.

**Table 1:** Demographic details and clinical assessments of the participants.

Categories	BCP (*n* = 19)	HCs (*n* = 19)	*p*-value
Age, mean ± SD, years	6.04 ± 2.52	6.10 ± 2.85	0.95
Gender, *n*	12 m, 7 f	13 m, 6 f	>0.99
GMFCS, *n*	I: 3; II: 5; III: 7; IV: 1; V:3	n/a	-
MACS, *n*	I: 5; II: 5; III: 7; IV:2	n/a	-
Motor distribution, *n*	Quadriplegia: 8; Diplegia: 11	n/a	-
Gestational age, *n*	<37 w: 15; ≥37 w: 4	≥37 w: 18; <37 w:1	-

*Abbreviations: BCP, bilateral cerebral palsy; f, Female; GMFCS, Gross Motor Function Classification System; HCs, healthy controls; m, Male; MACS, Manual Ability Classification System; n/a, Not applicable; SD, Standard deviation; w, Weeks*.

### MRI Acquisition

Each child was required to wear sponge earplugs for hearing protection. The subject’s head was immobilized using a moulded foam. Each child’s sleeping routine was adjusted to sleep soundly, reduce motion artifacts and ensure a complete MRI examination. The BCP children who were unable to remain still were sedated with chloral hydral (10%, 25–50 mg/kg) before the MRI examination. The underlying risk of the chloral hydrate was sufficiently explained to the participants’ guardians, and written informed consent was obtained. The HCs were required to keep their eyes closed and keep still without sedation. Respiration rate and heart rate were monitored throughout the whole process of MRI examination.

The same scanning protocol was used for both BCP and HC children as follows. T1-weighted 3D brain volume images were captured using a GE 3.0-T scanner (Signa HDxt, GE Healthcare, and Milwaukee, Wisconsin). High-resolution structural MR images were captured with the following scan parameters: echo times = 4.7 ms, inversion time = 400 ms, repetition time = 10.4 ms, slice thickness = 1 mm, gap = 0 mm, axial slices = 148, flip angle = 15°, and matrix size = 256 × 256. The head of the participants were immobilized using moulded foam. The selection, management, and monitoring of children were performed in compliance with previous guidelines (Alexander-Bloch et al., 2013; Cote and Wilson, 2016).

### MRI Preprocessing

Data preprocessing of T1-weighted images was performed using Statistical Parametric Mapping (SPM12) and Computational Anatomy Toolbox^1^ (CAT12) based on MATLAB^2^. Briefly, original T1 high-resolution anatomical images were first manually checked to ensure that there was no obvious artifact, incomplete scan, or poor contrast. These images were then format-converted and manually reoriented. The format-converted and reoriented T1 images were entered into the preprocessing pipeline in CAT12 with the default parameters. We applied a pediatric template (6–12 year-old) from the Imaging Research Center at Cincinnati Children’s Hospital Medical Center^3^. CAT12 can reconstruct the central surface and measure CT in one step. The spherical harmonic method was used to restore the topological flaws of cortical surface mesh (Fischl and Dale, 2000). Before the statistical analysis, the individual map of CT was smoothed by employing a Gaussian filter with a full-width at half-maximum of 15 mm (Fischl and Dale, 2000).

### Construction of Structural Covariance Network

**Figure 1:**
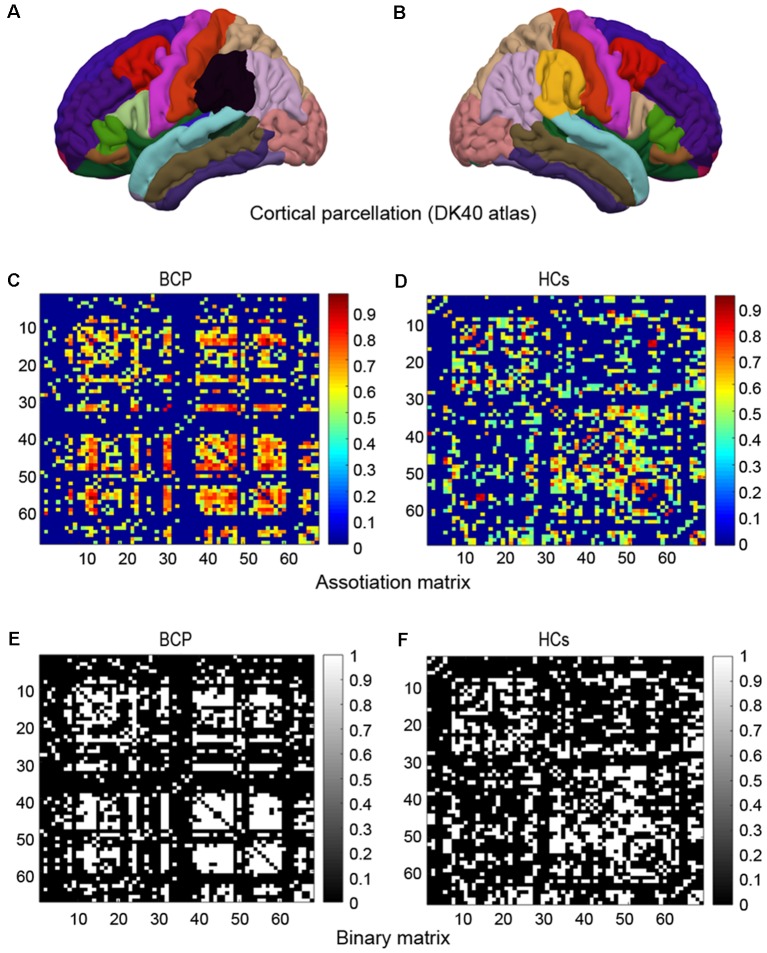
Cortical parcellation, association matrix and binary matrix. Cortical parcellation is according to DK40 atlas **(A,B)**. Correlation matrices for the bilateral cerebral palsy (BCP; **C**) and healthy controls (HCs; **D**). The color-bar shows the strength of the connections. Binary adjacency matrices for the BCP **(E)** and the HCs **(F)**, with white indicating the presence of connection and black the absence of one.

We constructed a SCN based on an interregional CT Pearson’s correlation matrix for interregional CT values. Network nodes were defined by a classic cortical parcellation scheme according to Desikan-Killiany Atlas (Figures 1A,B; Destrieux et al., 2010), which is an automated recognition system for dividing the cerebral cortex into 68 gyral-based regions of interest (ROIs). The Desikan-Killiany Atlas with 68 parcels bilaterally (DK40 atlas) is often used in the construction of SCNs. The Graph-Theoretical Analysis Toolbox (GAT) was used to perform the graph analysis (Hosseini et al., 2012). Edges were identified by the inter-areas Pearson’s correlation of CT, which was acquired from each individual surface. The strength of the edges of the covariance network was defined by using Pearson’s correlation between the cortical thicknesses of each pair of brain regions across all subjects.

A 68 × 68 association matrix M was established for each group. The association matrix was obtained by calculating the inter-area Pearson’s correlation coefficients (r_ij_) between the CT of every pair of ROIs “i” and “j” (Figures 1C,D). Then the association matrix was thresholded into binary matrix A with values of 1 or 0 (Figures 1E,F). The thresholds were set across a density range of network (Dmin = 0.11 and Dmax = 0.45). The Dmin denotes the minimum density that would be able to allow the networks to be adequately connected (Hosseini et al., 2012). We evaluated both regional and global network parameters across the range of network densities (i.e., 0.11–0.45) at an interval 0.02. Then, by considering matrix A to be graph G, we restricted the quantities N, K, D, i, j, and k as the total number of ROIs (i.e., 68), number of edges, percentage of surviving edges at a specific density threshold, and three randomly selected nodes, respectively. For a detailed description, see Hosseini et al. (2012).

### Network Measures

Global network measures included clustering, path length, small-worldness, and global efficiency; local network metrics including betweenness, degree, and local efficiency were calculated.

Briefly, the small-worldness (a metric reflecting the degree of network economic optimization) was identified as a combination of a high clustering (a metric reflecting the degree of local segregation) and a short path length (a metric reflecting efficient communication of the whole brain network; Watts and Strogatz, 1998), i.e., the ratio of normalized clustering (the ratio of local clustering real network and mean clustering of 20 random networks) and normalized path length (the ratio of characteristic path length of real network and mean path length of 20 random networks). The b_i_ is defined as the number of shortest paths that pass-through node i. The k_i_ denotes the sum of edges that run through node i and other nodes. The bi and ki denote the mean betweenness and degree of the entire network, respectively.

For the small-worldness index, we analyzed the parameters both at Dmin and across the density range (0.11–0.45) using area under the curve (AUC). A network was regarded as small-world when the ratio at Dmin was >1 (Watts and Strogatz, 1998). In addition, the hub of the network was discerned by b_i_ and k_i._ The node was identified as a hub when regional value was at least 2 SDs larger than the mean value. For a detailed description of the above-mentioned measures and mathematical formulas, please refer to (Watts and Strogatz, 1998; Lerch and Evans, 2005; Bassett and Bullmore, 2006; Bullmore and Bassett, 2011), and our recent publications (Jiang et al., 2016, 2018).

To explore the vulnerability of the SCNs to targeted attacks and random failures, we analyzed the mass of the maximum persevering component in response to the successively random or targeted removals of nodes. First, we removed one node from the SCNs at random, and then measured the variation in the size of the residual giant component. Second, we randomly selected and removed additional nodes from the SCNs and recomputed this measure. The above procedures were performed for the SCNs of both BCP and control groups, and a comparison of the results was further investigated.

### Statistics

For the CT maps, general linear modeling, installed in the CAT, was used to perform vertex-wise group inference on the smoothed cortical surfaces (Bullmore and Bassett, 2011; Luo et al., 2015). These included: (1) vertex-wise CT changes between the BCP and controls (independent samples Student’s *t*-test); and (2) correlations with MACS (multiple regression). These results were corrected for multiple comparisons by threshold-free cluster enhancement (TFCE) with 10,000 permutations and cluster-level family-wise error (FWE) *p* < 0.05.

For the significance of the differences in topological metrics between groups, a nonparametric permutation test method was applied. Inter-group comparisons were made with the GAT toolbox and *p* < 0.05 with false discovery rate (FDR) correction was considered statistically significant.

## Results

### Demographics

We recruited 19 children with BCP, including seven girls and 12 boys, with mean ± SD age of 6.04 ± 2.52 years. Nineteen demographically matched HCs, including six girls and 13 boys, with a mean ± SD age of 6.10 ± 2.85 years were included.

Demographic details and clinical assessments are listed in Table 1.

### Between-Group Comparison of CT

**Figure 2:**
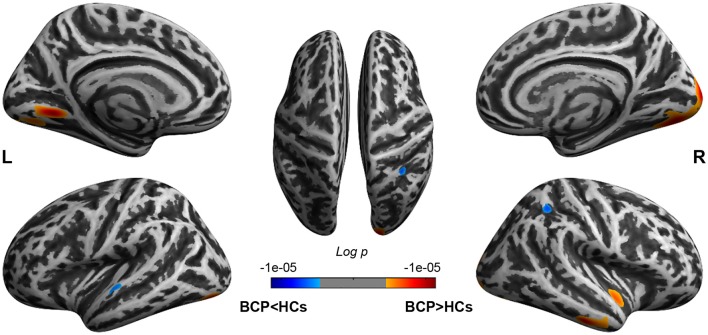
Brain regions show CT differences between BCP and HCs. The thicker cortices are observed in the bilateral hemispheres (lateral occipital, lingual, fusiform), and right hemisphere (cuneus, peri calcarine, inferior temporal, middle temporal, superior temporal, insula). The thinner cortices are observed mainly in the left superior temporal and right superior parietal regions. CT, cortical thickness; BCP, bilateral cerebral palsy; HCs, healthy controls; L, left; R, right.

The significant CT clusters were projected onto the template brain surfaces (Figure 2, Table 2). Compared with the HCs, children with BCP displayed significantly thicker cortices in the bilateral hemispheres (lateral occipital, lingual, fusiform), and the right hemisphere (cuneus, peri calcarine, inferiortemporal, middle temporal, superior temporal, insula), and significantly thinner cortices in the left superior temporal and right superior parietal regions (*p* < 0.05, FWE corrected).

**Table 2:** Results of group comparison of cortical thickness (CT) between BCP and HC groups.

Contrast		Cortical area	Size	*p*-value
BCP > HCs	Left	100% lingual	875	0.00001*
		91% lateral occipital, and 9% fusiform	586	0.00022*
		86% lingual, and 13% fusiform	462	0.00066*
	Right	48% lateral occipital, 24% lingual, 14% fusiform, 7% cuneus, and 6% pericalcarine	3,441	0.00001*
		56% inferior temporal, and 44% middle temporal	724	0.00006*
		86% superior temporal, and 14% insula	519	0.00037*
BCP < HCs	Left	100% superior temporal	234	0.00118*
	Right	100% superior parietal	377	0.00044*

*Based on Desikan-Killiany Atlas with 68 parcels bilaterally. BCP, Bilateral cerebral palsy; HCs, Healthy controls.

### Between-Group Differences in Global Network Measures

#### Global Differences Across Network Densities

**Figure 3:**
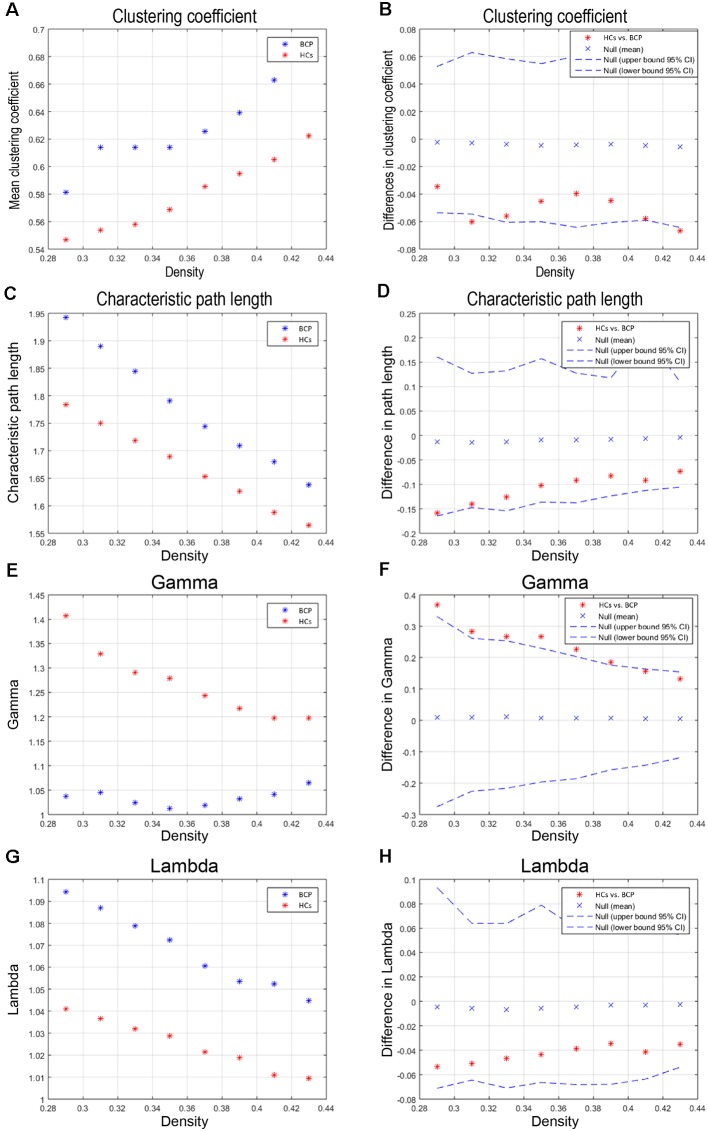
Within-group global network measures, and between-group differences in these measures. Clustering coefficient **(A,B)**, Characteristic path length **(C,D)**, Gamma **(E,F)**, and Lambda **(G,H)** of the BCP and HC networks. The red star indicates the difference between the two groups **(B,D,F,H)**. The value of gamma is significantly lower in BCP group. BCP, bilateral cerebral palsy; HCs, healthy controls.

**Figure 4:**
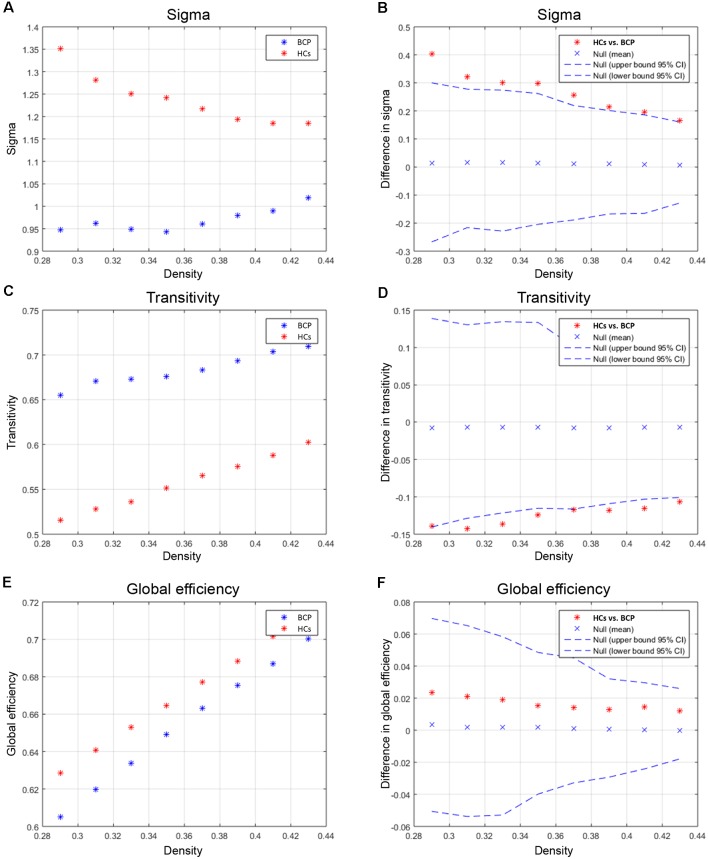
Between-group differences of global measures, sigma **(A,B)**, transitivity **(C,D)** and global efficiency **(E,F)**. There are significant between-group differences in sigma **(B)** and transitivity (**D**; *p* < 0.05, FDR-corrected). The value of sigma is lower in BCP group. The value of transitivity is higher in BCP group. BCP, bilateral cerebral palsy.

We compared the differences in characteristic path length, clustering coefficient, gamma, and lambda of the BCP and HC networks (Figure 3). The value of gamma was significantly lower in BCP group (Figure 3F). We identified inter-group differences in global measures at various densities (Figure 4). The value of sigma was significant lower in BCP group (*p* < 0.05, FDR-corrected; Figure 4B). The value of transitivity was significantly higher in the BCP group (*p* < 0.05, FDR-corrected; Figure 4D).

#### AUC Analysis of Global Network Measures

We then contrasted inter-group differences of parameters (normalized clustering coefficient, normalized degree, and normalized node betweenness) across a density range of network (i.e., AUC results). We compared the AUC between the two groups. The red star shows the change between the two groups. All regions survive following FDR correction (*p* < 0.05).

**Figure 5:**
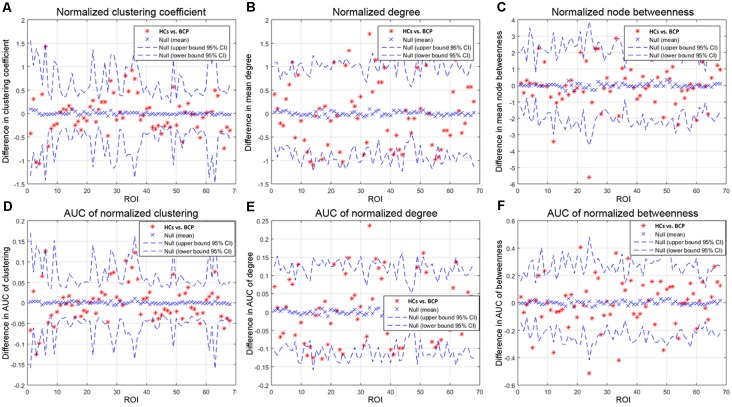
Between-group differences of measures [normalized clustering coefficient **(A)**, degree **(B)**, and node betweenness **(C)**] across a range of network densities (i.e., AUC results). The red star indicates the difference between the two groups. All regions survive following FDR correction (*p* < 0.05). The BCP group exhibits a significantly different AUC for network metrics [normalized clustering **(D)**, *p* = 0.02; small-worldness **(E)**, *p* = 0.02; transitivity **(F)**, *p* = 0.04] as compared with the HCs. BCP, bilateral cerebral palsy. AUC, area under the curve.

Children with BCP showed significantly decreased normalized clustering coefficient in right hemisphere (superior temporal, transverse temporal; Figure 5A); significantly increased normalized degree in right medial orbitofrontal and left pars opercularis (Figure 5B); significantly decreased normalized node betweenness in right hemisphere (fusiform, lingual; Figure 5C); significantly decreased AUC of normalized clustering in right superior temporal and left insula (Figure 5D); significantly increased AUC of normalized degree in right medial orbito frontal and left hemisphere (pars opercularis, parsorbitalis rostral middle frontal), and significantly decreased AUC of normalized degree in left hemisphere (inferior parietal, isthmus cingulate; Figure 5E); significantly increased AUC of normalized betweenness in left hemisphere (lateral orbitofrontal, pars opercularis, pars triangularis), and significantly decreased AUC of normalized betweenness in left hemisphere (caudal middle frontal, rostral anterior cingulate) and right hemisphere (fusiform, lingual, pars opercularis; Figure 5F).

### Network Hubs

We identified the hub nodes based on two measures, betweenness and degree, respectively.

#### Betweenness Based Hub Identification

The common hubs in the two groups contained the bilateral precuneus. Hubs specific to BCP were found in the left inferior parietal, left isthmus cingulate, left paracentral, left postcentral, left superior parietal, right fusiform, right lingual, right paracentral, and right superior parietal regions. Network hubs specific to HCs were identified in the left cuneus, left lateral-orbitofrontal, left medial-orbitofrontal, left pars opercularis, left rostral-middle frontal, right medial-orbitofrontal, and right transverse-temporal regions. The BCP networks showed a displacement of hubs to the left parietal and right occipital lobes and a reduction in the left frontal lobe.

#### Degree Based Hub Identification

In both groups, the common hubs included the left medial-orbitofrontal and right precentral areas. Hubs specific to the BCP group were found in the left caudal-middle-frontal, left rostral-anterior-cingulate, right fusiform, right lingual, right pars opercularis, and right superior-frontal areas. Hubs specific to HCs were identified in the left cuneus, left lateral-orbitofrontal, left lingual, left middle temporal, left pars opercularis, left pars triangularis, left precentral, left precuneus, left rostral-middle-frontal, right inferior-temporal, right medial-orbitofrontal, right precuneus and right transverse-temporal areas.

### Between-Group Differences in Regional Network Measures

#### Regional Differences Across Network Densities

We compared the regional metrics between the two groups (Table 3).

**Table 3:** Between-group differences in regional network measures.

	BCP < HCs	BCP > HCs	*p*-value
Degree	l pars orbitalis	l isthmus cingulate	0.01
	l pars opercularis		
	l rostral middle frontal		
Clustering coefficient	l pars opercularis	l cuneus	0.01
		r postcentral
		r transverse temporal	
Local efficiency	l pars opercularis	r caudal anterior cingulate	0.01
Node betweenness	l entorhinal	r fusiform	0.01
	l pars opercularis	r lingual
	l pars triangularis	l caudal middle frontal	
		l rostral anterior cingulate	

*Abbreviations: BCP, Bilateral cerebral palsy; HCs, Healthy controls; l, left; r, right*.

**Figure 6:**
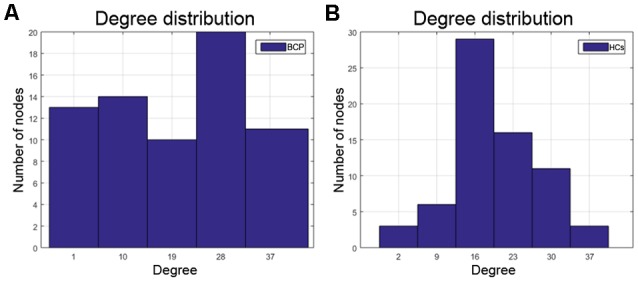
The degree distribution in BCP is non-normal **(A)**, which may represent a kind of disorder. The degree distribution of HCs is relatively normal **(B)**. BCP, bilateral cerebral palsy; HCs, healthy controls.

Degrees of regions, containing the left pars orbitalis, left pars opercularis, and left rostral middle frontal areas, are significantly smaller for the BCP group, while the left isthmus cingulate showed a significantly greater degree in the BCP group. The degree distribution in BCP is non-normal (Figure 6A). The degree distribution in HCs is normal (Figure 6B).

For regions of clustering coefficient and local efficiency, the left pars opercularis exhibited significantly smaller metrics in the BCP group. Regions of clustering coefficient, including the left cuneus, right postcentral, and the right transverse temporal, were significantly greater for the BCP group. As for regions of local efficiency, only the right caudal anterior cingulate showed significantly greater efficiency in the BCP group. Regions of node betweenness, including the left entorhinal, left pars opercularis, and left pars triangularis, were significantly smaller for the BCP group; other regions, including the right fusiform, right lingual, the left caudal middle frontal, and left rostral anterior cingulate, were significantly greater for the BCP group.

All above regions survived after FDR correction (*p* < 0.05).

### Random Failure and Targeted Attack Analysis

**Figure 7:**
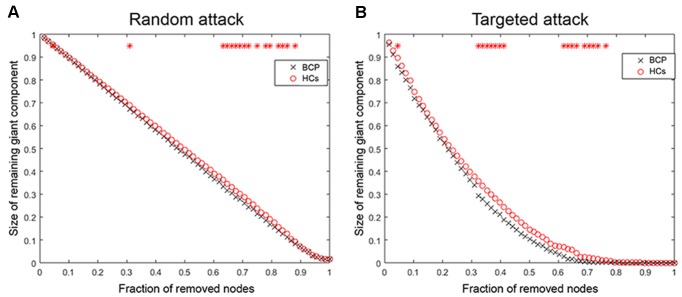
Random and targeted attack analysis. Alterations in the size of the largest preserving component of the network are shown as a function of a fraction of randomly **(A)** and targeted **(B)** removed nodes. In many proportions of the deleted nodes and the AUC results, there was no significant difference between the two groups (*p* > 0.05) of the resilience of the networks to both targeted and random attacks. AUC, area under the curve. BCP, Bilateral cerebral palsy.

We analyzed the network resilience in response to random (Figure 7A) and targeted (Figure 7B) attacks. We found that the network resilience of BCP children showed a significant reduction (under several network densities) in response to both targeted and random attacks, although not under all network densities (through the AUC curve). Variation in the size of the largest preserving component of the network is shown as a function of a fraction of targeted and randomly removed nodes. In lots of deleted nodes and AUC results, there is no significant different in the resilience of the network to random failure and targeted attacks in the two groups (*p* > 0.05).

The network of BCP shows less robustness to the targeted attack as compared with HCs, but the statistical significance was only identified at a few fractions of deleted nodes (*p* < 0.05, the red star indicates that there is a significant difference across different network densities between the two groups).

### Correlation Analyses

We found the CT in the right superior and inferior temporal cortices were significantly negatively correlated with hand function (vertex *p* < 0.001, cluster level correction).

## Discussion

In this study, we investigated differences in CT and SCNs between BCP children and HCs to identify the effects of brain lesions on the developing brain. Specifically, BCP children showed: (1) aberrant foci of CT, obviously increased thickness in the bilateral occipital areas, right temporal and insular areas, and reduced thickness in the left superior temporal and parietal areas; and (2) altered network configurations, including sub-optimized small-worldness, reduction of local segregation, changes in the layout of densely connected hub nodes, and reduced network resilience to random and targeted attacks. These results suggest abnormal cortical neuroanatomy foci and network connectivity patterns in children with BCP, particularly in the brain system of visual, linguistic, and multi-dimensional sensory integration. Abnormal CT and its structural wiring may be the neuroanatomical basis of BCP.

The comparison results of CT are basically consistent with previous studies (Hagmann et al., 2008; Scheck et al., 2014). The lateral occipital, the left superior temporal gyrus, and the right superior temporal and parietal areas are mainly involved in the integration of visual, linguistic comprehension and multi dimensional sensory information. Although we did not find a significant association between the hand function classification in children with BCP and CT in primary sensorimotor and supplemental motor areas, we found significant negative correlations between the CT in the right temporal lobe and hand function. Future studies should increase the sample size and improve the homogeneity of the sample and hopefully will find an association among sensory motion areas. These results are also consistent with the clinical symptoms of children with BCP.

Beyond the cross-sectional analysis of abnormal CT foci in BCP, this study extended the SCN analysis on this basis. The recent decade has witnessed the development of the human brain connectome. The human brain is modeled as complexly connected network architecture with a topological hierarchy. For the global measures, we found that the small-worldness of SCNs in children with BCP was significantly reduced, both under the Dmin threshold and the AUC curve across multiple network densities. This reduction suggests that children with BCP are sub-optimized relative to typical development HCs, without an optimal balance between segregation and integration, and cost and efficiency (Watts and Strogatz, 1998; Bullmore and Sporns, 2009; Alexander-Bloch et al., 2013). Further analysis found that the normalized clustering coefficients were significantly reduced and normalized path length was increased, contributing to a significant reduction in small-worldness attributes. Using various morphological metrics to build SCNs, past researches have shown small-world architecture in brain networks during typical development in HCs (Fan et al., 2011). Through an optimal balance between integration and segregation, this kind of SCNs enables efficient information processing. Therefore, children with BCP are more likely to have abnormalities in local segregation; that is, there are more local defects.

For the local measures of SCNs, including clustering coefficient, nodal degree, local efficiency and betweenness, significant decreases were observed in the left pars opercularis and dorsolateral prefrontal areas, and significant increases were observed in the right anterior cingulate, right postcentral, occipital, and Heschl’s areas. These alterations are also involved basically in functions such as speech, execution, and language comprehension.

Children with BCP showed significant decreased normalized clustering coefficient in right hemisphere (superior temporal, transverse temporal; Figure 5A), and significant decreased AUC of normalized clustering in right superior temporal and left insula (Figure 5D). Small-world topology is characterized by high efficiency (clustering coefficient) with a low wiring cost (path length; Bullmore and Sporns, 2009), which enables effective integration of multiple segregated sources of information. Therefore, decreased clustering coefficient may indicate that the information segregation and integration in BCP children are both compromised. Such deficits may underlie the cognitive inabilities, such as visuo-spatial in BCP children.

Children with BCP showed significant increased normalized degree in right medial orbito frontal and left pars opercularis (Figure 5B). Children with BCP showed significant decreased normalized node betweenness in right hemisphere (fusiform, lingual; Figure 5C). Children with BCP showed significant increased AUC of normalized betweenness in left hemisphere (lateral orbitofrontal, parsopercularis, pars triangularis), and significant decreased AUC of normalized betweenness in left hemisphere (caudal middle frontal, rostral anterior cingulate) and right hemisphere (fusiform, lingual, pars opercularis; Figure 5F). Node betweenness is a measure of the centrality of a node in a graph, and group differences in betweenness reflect the effects of the BCP disease on the global roles of the affected regions in the cortical network. Our study showed changes of node betweenness in some core regions of DMN. A possible explanation relies on a fact that such regions in present study serve to link different functional modules in the brain, such as executive network (superior frontal) and visual network (cuneus), and deficits of such cognitive ability has been reported in BCP children.

We further analyzed the densely-connected nodes, also known as hubs, and their layouts and changes. The hub node is generally considered to be the infrastructure of the human brain network, supporting a highway for network information integration and segregation (Mišić et al., 2015). Hubs comprise a very small proportion compared to non-hub nodes and are therefore less susceptible to attack. Previous meta-analysis indicated that some major brain diseases are due to damage in the hubs (Crossley et al., 2014). In this study, we found that the number of hubs in children with BCP increased and the anatomical location also shifted. Consistent with previous studies (Hagmann et al., 2008; van den Heuvel and Sporns, 2013), we found that the hub nodes of typical developed children are mainly located in the transmodal and multimodal association cortices, especially in the regions of the default mode and executive control networks (Raichle, 2015). However, the hub nodes of children with BCP appear in areas of sensorimotor and unimodal sensory association. These results suggest that the network core configuration of BCP patients shifts from the association cortex to the primary and unimodal association.

We finally analyzed the network resilience and degree distribution. We found that SCNs in children with BCP showed a significant reduction (under several network densities) in response to both random and targeted attacks, although not at under all network densities. These results suggest that SCNs in BCP children present a relatively fragile topology that may present more severely fatal and catastrophic consequences in response to nodal damage than HCs. In addition, the results of the degree distribution showed that the nodal degree of BCP had a similar “average” distribution, while the healthy development of typical developmental children followed a “power-law” distribution, which is consistent with previous studies (Barabasi and Albert, 1999; Hart et al., 2016). These results reflect that the node topology role differentiation of children with BCP is not successful enough.

There are several limitations in this study. First, this is a cross-sectional study and cannot show a causal relationship of CT and hand function in CP children, even if a significantly negative association was found between CT and manual ability. Thus, whether there is a causal relationship deserves further investigation using the longitudinal and prospective design. Second, some clinical data (such as education, hormone level, and treatment history) and behavioral and environmental factors (life-style, physical exercise, and dietary patterns) are crucial factors that may impact the CT and hand function. Unfortunately, we did not assess these potential and crucial factors in this study, which should be evaluated in future research. Third, the small sample size may have reduced the ability to detect an effect across groups.

## Conclusion

In summary, this study analyzed a comprehensive view of CT changes and graph analysis of CT-based structural connectivity in children with BCP. It revealed abnormal topological organization of SCNs. The present study found that foci (right superior temporal and subtemporal cortex) with significant negative relationships between the CT and hand function, suggesting the uncoordinated development of patterns of transmodal areas. It also provides evidence for the concept that BCP is a disorder that features an abnormal CT and the uncoordinated development of structural wiring, disrupted nodal profiles of betweenness, and shifted hub distribution that may represent a neuroanatomical hallmark of BCP in the developing brain. The observed changes may not only reflect neurodevelopmental aberrations but also compensatory mechanisms.

## Data Availability Statement

All datasets generated for this study are included in the manuscript.

## Ethics Statement

This study was approved by the institutional review board of the First Affiliated Hospital of Xi’an Jiaotong University and was registered on the ClinicalTrials.gov registry (Identified: NCT02979743). This study was approved by the medical ethics committee and was conducted in accordance with the Declaration of Helsinki. Verbal and written informed consent was obtained from the subjects’ parents or guardians.

## Author Contributions

JY and TZ designed the experiments. HL, HJ, MW, XW, HZ, YC, XT, CL, and TH performed the experiments. HL, WB, XL, and CJ analyzed the data. HL, HJ, and WB wrote the manuscript. JY, TZ, and BH revised the manuscript.

## Conflict of Interest

The authors declare that the research was conducted in the absence of any commercial or financial relationships that could be construed as a potential conflict of interest.

## Footnotes

^1^www.neuro.uni-jena.de/cat/

^2^www.mathworks.com/

^3^https://irc.cchmc.org/

## Abbreviations

BCP: bilateral cerebral palsy
SCNs: structural covariance networks
CT: cortical thickness
HCs: healthy controls
AUC: area under the curve
MACS: manual ability classification system
FDR: false discovery rate
PWMI: periventricular white matter injury
MRI: magnetic resonance imaging
PVL: periventricular leukomalacia
GMFCS: gross motor function classification system
CAT: computational anatomy toolbox
ROIs: regions of interest
GAT: graph-theoretical analysis toolbox.
